# CLEC12A-directed immunocytokine with target cell–restricted IL-15 activity for treatment of acute myeloid leukemia

**DOI:** 10.3389/fimmu.2025.1561823

**Published:** 2025-03-27

**Authors:** Boris Klimovich, Leonard Anton, Jinwon Jung, Yangmi Lim, Bora Lee, Jonghwa Won, Latifa Zekri, Anna Chashchina, Martin Pflügler, Jonas S. Heitmann, Gundram Jung, Helmut R. Salih

**Affiliations:** ^1^ Clinical Collaboration Unit Translational Immunology, Department of Internal Medicine, University Hospital Tübingen, Tübingen, Germany; ^2^ Cluster of Excellence iFIT (EXC 2180) ‘Image-Guided and Functionally Instructed Tumor Therapies’, Eberhard Karls University of Tübingen, Tübingen, Germany; ^3^ ABL Bio Inc., Seongnam-si, Gyeonggi-do, Republic of Korea; ^4^ Department of Immunology, Institute for Cell Biology, Eberhard Karls Universität Tübingen, Tübingen, Germany; ^5^ German Cancer Consortium Deutsche Konsortium für Translationale Krebsforschung (DKTK), partner site Tübingen, a partnership between Deutsche Krebsforschungszentrum (DKFZ) and University Hospital Tübingen, Tübingen, Germany

**Keywords:** IL-15, NK cells, AML, immunocytokines, Fc-optimized antibodies, Clec12A, CLL-1

## Abstract

Despite recent advancements, acute myeloid leukemia (AML) remains a therapeutic challenge. While monoclonal antibodies (mAbs) leveraging natural killer (NK) cells through antibody-dependent cellular cytotoxicity show great potential, none have gained clinical approval for AML. Immunocytokines have emerged as a promising strategy to overcome the limited efficacy of therapeutic antibodies. IL-15 stimulates activation, proliferation cytotoxic activity of NK cells, but its clinical use is prevented by short half-life, poor accumulation in the tumor, and toxicity due to systemic off-target immune activation. Here we report on the generation and preclinical characterization of modified immunocytokines consisting of an Fc-optimized CLEC12A (CLL-1) antibody fused to an IL-15 moiety with E46K mutation. The mutation abrogates binding to IL-15Rα, thereby enabling substitution of physiological trans-presentation by target binding and thus conditional IL-15Rβ/γ stimulation to reduce systemic toxicity. An optimal CLEC12A binder was selected from a range of murine mAbs, based on analysis of AML cell lines and leukemic cells from patients. This antibody was then used to construct an immunocytokine (MIC12) that subsequently was characterized functionally. Analysis of NK cell activation, cytokine release, proliferation and anti-leukemia reactivity demonstrated that MIC12 induced superior target cell killing and NK cell expansion compared to Fc-optimized CLEC12A antibody, with efficacy being dependent on target antigen binding. Our results show that novel immunocytokines with conditional IL-15 activity are capable of inducing potent NK cell responses against AML cells and identify MIC12 as promising therapeutic candidate for leukemia treatment.

## 1 Introduction

Acute myeloid leukemia (AML) is an aggressive disease characterized by clonal expansion of myeloid progenitor cells with impaired differentiation (1). It is the most common form of acute leukemia in adults, with a median age at diagnosis of 68 years (2). Although various immunotherapeutic approaches such as immune checkpoint inhibitors (3), chimeric antigen receptor (CAR) T cells (4) or bispecific antibodies (5) are currently evaluated in clinical trials, the standard of care for AML patients remains chemotherapy, targeted therapy with small molecules and allogeneic stem cell transplantation. In particular elderly patients face a poor prognosis and a high risk of relapse (6). This emphasizes the pressing need for new therapeutic targets and advanced treatment options.

Monoclonal antibodies (mAb) have revolutionized the treatment of several types of cancer. Rituximab, the first clinically available mAb targeting CD20, and further optimized constructs directed to CD20 have become a mainstay in the treatment of B-cell lymphomas. Despite their remarkable efficacy in lymphoid malignancies, attempts to develop effective mAbs for treating AML were so far not overly successful (7). Efficacy of Rituximab is largely driven by induction of antibody-dependent cellular cytotoxicity (ADCC) mediated by NK cells (8, 9). Enhancing mAb affinity for FcγRIIIa/CD16a through Fc domain modifications, including glycoengineering (10) or mutagenesis, is a common strategy to strengthen ADCC-inducing capabilities. A common example of the latter are the mutations S239D and I332E (hereafter referred to as SDIE modification) (11, 12). As an example, a SDIE-optimized mAb targeting FLT3 in patients with AML in complete remission with persisting minimal residual disease of AML has demonstrated promising results in a Phase I clinical trial (13). However, achieving remission in AML patients with high leukemic burden requires to further enhance mAb efficacy in stimulating NK cells, and combining mAbs with immunostimulatory cytokines as so called immunocytokines offers an attractive approach. Particularly IL-2 and IL-15 are promising in this regard, since they not only boost anti-leukemic activity but also promote NK cell proliferation, which is essential for combating larger tumor burden (14–18). However, the administration of recombinant cytokines is associated with severe side effects, and this would also hold true upon combined application with mAbs. Likewise, most immunocytokines developed so far face the same fundamental problem: in contrast to mAbs, their cytokine moiety lacks conditional target-restricted activity, as the cytokine moieties act independently of target binding. As a consequence, off-target activation of immune cells leads to substantial side effects that constrain dosing and thereby reduce efficacy (19–21).

The unique mechanism of action of IL-15 offers a solution to this fundamental issue: IL-15 effectively engages the IL-15Rβ/γ receptor on cytotoxic lymphocytes only when presented *in trans* upon binding to the IL-15Rα chain expressed on monocytes and dendritic cells (22–24).

Our group recently introduced a conceptually novel format of target cell-restricted IL-15- “modified immunocytokines” (MIC), designed to deliver conditional cytokine activity to effector cells after the antibody component binds to its target (25). MIC are composed of an SDIE-optimized mAb fused to an IL-15^E46K^ mutein with abrogated IL-15Rα interaction (26). Antibody engagement with the target replaces the binding to IL-15Rα and then conditionally stimulates IL-15Rβ/γ-expressing effector cells. MICs directed against CD20 and CD19 were shown to be superior to Fc-optimized antibodies in inducing NK cell-mediated ADCC and displayed encouraging efficacy in preclinical models while producing substantially weaker off-target effects than classical IL-15-immunocytokines (25).

To extend this strategy to the therapy of AML we developed MIC directed against the CLEC12A antigen (CLL-1). CLEC12A is reportedly expressed on leukemic cells in 90% of AML cases but absent on healthy hematopoietic stem cells (27, 28). This expression pattern makes CLEC12A an attractive target for AML immunotherapy as exemplified by a large body of preclinical data and ongoing clinical trials (29). We here characterized 3 different CLEC12A antibodies to identify an optimal target binder, generated an Fc-optimized MIC construct and functionally characterized it with regard to efficacy using cell lines and primary AML cells.

## 2 Material and methods

### 2.1 Antibodies and immunocytokines

Sequences of murine CLEC12A mAbs 16C6, 33C2 and 84A2 were obtained from ABL Bio. Humanization was performed by CDR grafting. Variable domains of heavy and light chains were synthesized as double-strand DNA fragments (Thermo Fischer Scientific) and cloned in frame with Igγ1 or Igκ1 constant regions respectively downstream of the CMV promoter. Vectors carrying wild-type or optimized (S239D and I332E mutations) Fc domains were used for corresponding constructs. MIC constructs were generated by fusing the IL-15E46K mutein to the C-terminus of the heavy chain using a (G_4_S)_4_ linker, a widely adopted flexible and low-immunogenic linker that has been validated in several clinical-stage biologics.

Plasmids were purified using NucleoBond Xtra Maxi kit (Macherey-Nagel). Antibodies were produced in Expi-CHO expression system (Thermo Fischer Scientific) according to the manufacturer’s instructions. Purification from culture supernatants was conducted via affinity chromatography on Protein A Sepharose (Mabselect; Cytiva) and subsequent size exclusion chromatography (HiLoad 16/60 Superdex200; Cytiva). Subsequently, analytical size exclusion chromatography (Superdex 200R PC3.2/30 column; Cytiva) and SDS-PAGE in 4-12% gradient gels (Invitrogen) were performed to assess antibody size, aggregation and integrity. Endotoxin content was determined using Endonext Endozyme II (Biomérieux). Preparations containing less than the detection limit of the assay (0.001 EU endotoxin) were considered endotoxin free and used in experiments. When necessary, the Endotrap HD kit (Lionex) was used to remove endotoxins.

To account for differences in molecular weight, equimolar concentrations of MIC and Fc-optimized mAb 1,2 µg/ml and 1 µg/ml, respectively were used if not otherwise indicated.

### 2.2 Cell culture

Peripheral blood mononuclear cells (PBMC) of healthy donors and AML patients were isolated by FICOLL density gradient centrifugation (Pancoll human; PAN-Biotech) and frozen in liquid nitrogen. Informed consent was obtained from all patients and healthy donors in accordance with the Helsinki protocol and the guidelines of the local ethics committee (approval number 13/2007V).

Isolation of human NK and T cells was performed using the human NK cell isolation kit and the human pan T cell isolation kit (Miltenyi Biotec) according to the manufacturer’s instructions.

Human cell lines U937, EOL-1, TF-1 and M07e were obtained from German Collection of Microorganisms and Cell Cultures (Deutsche Sammlung von Mikroorganismen und Zellkulturen, DSMZ) and cultured in RPMI 1640 medium (Thermo Fischer Scientific) supplemented with 10% heat-inactivated fetal bovine serum (PAN-Biotech) and 1% penicillin-streptomycin (Lonza). Cultures of TF-1 and M07e cells were supplemented with 2 and 10 ng/µl GM-CSF (Miltenyi Biotec) respectively.

The respective immunophenotype described by the provider was validated after thawing using flow cytometry. Culture supernatants were monthly PCR-tested for mycoplasma contamination.

U937 cells were labeled with GFP using lentiviral infection. A second-generation lentiviral vector containing the EGFP-T2A-Puro cassette under the CMV promoter was cloned by Vector Builder. Lentiviral particles were produced by co-transfecting HEK293T cells with the vector and packaging plasmids (psPAX2 and pMD2.G, Addgene #12260 and #12259, gifts from Didier Trono) using a standard calcium-phosphate transfection protocol. The lentiviral supernatants were then concentrated using the Lenti-X concentrator (Takara) according to the manufacturer’s instructions. U937 cells were infected with the concentrated lentiviral particles via spinoculation (1 hour at 600g, 37°C) and selected with 1 µg/ml puromycin.

### 2.3 Flow cytometry

CLEC12A expression and binding characterization of CLEC12A antibodies were determined by incubation of CLEC12A-expressing cells with the indicated constructs or isotype controls, followed by goat-anti-mouse-PE or donkey-anti-human PE conjugates (Jackson ImmunoResearch Europe Ltd) staining for murine and human antibodies respectively and analyzed using flow cytometry. BD LSRFortessa (BD Biosciences) was used for data acquisition, data analysis was performed in FlowJo v9 (FlowJo LCC). The following fluorescent antibody conjugates were used: CD3-APC-Fire 750 (SK7, Biolegend), CD33-APC (WM53, Biolegend), CD34-APC (clone 581, BD Bioscience), CD38 and CD56-PE/Cy7 (HCD56, Biolegend); life-dead staining was performed using 7-aminoactinomycin (7-AAD) (Biolegend).

Leukemic cells in patient samples were first selected by FSC/viability gating (staining with 7-AAD (BD Biosciences) and then gates were applied dependent on surface expression of CD33, CD34 or CD38. Gating strategy is shown in **Supplementary Figure S4**. Specific fluorescence intensity (SFI) were calculated by dividing mean fluorescence intensities measured with specific antibody by mean fluorescence intensities obtained with isotype control.

### 2.4 Human NK and T cell activation and proliferation

To analyze NK and T cell activation, upregulation of CD69 on the surface of CD3^-^CD56^+^, CD3^+^CD4^+^ and CD3^+^CD8^+^ subpopulation was analyzed by flow cytometry using an CD69-PE (FN50, BD Bioscience) staining.

For analysis of proliferation, PBMC of healthy donors were labelled with CellTrace™ Violet Cell Proliferation Kit (CTV) according to the manufacturer’s instructions (Thermo Fisher). After 6 days of co-incubation with target cells and respective antibodies, dye dilution was analyzed by flow cytometry in NK and T cells identified by counterstaining for CD3, CD4, CD8 and CD56.

### 2.5 Cytokine quantification

IFN-γ secretion was quantified using enzyme-linked immunosorbent assay (ELISA). For ELISA anti-human IFN-γ monoclonal antibody clones 2G1 and biotinylated B133.5 (Thermo Fisher), as well as poly-HRP 20 Streptavidin (Fitzgerald) were used. The TMB 2-Component Microwell Peroxidase Substrate Kit (Seracare) was used as substrate.

### 2.6 Cytotoxicity assays

For analysis of NK cell-mediated cytotoxicity, target cells were labelled with CTV. After co-incubation with PBMC of healthy donors and the indicated treatment, living target cells (CTV^+^7-AAD^-^) were quantified using flow cytometry. Analysis of equal assay volumes was ensured by using standard calibration beads (3 µm latex beads; Sigma-Aldrich).

Co-cultures of primary AML samples were supplemented with 50 ng/ml G-CSF, IL-3, IL-6, TPO, Flt3L and 100 ng/ml SCF (Peprotech). Due to the higher resistance of primary AML cells to NK cell-mediated lysis compared to cell lines, effector-to-target ratios were adapted based on preparative titration experiments.

Long-term cytotoxicity was analyzed with the IncuCyte S3 Live-Cell Analysis System (Sartorius). GFP-expressing U937 cells were co-cultured with PBMC of healthy donors and treated as indicated. Cells were imaged every 4h and after Top-Hat background subtraction, living target cells were identified with a predefined mask (size and fluorescence intensity) and counted.

### 2.7 Statistical analysis

GraphPad Prism 9 was used to generate all plots and perform statistical analysis. Statistical analyses included the Friedman test with Dunn’s multiple comparison test for comparing related groups, the Kruskal-Wallis test with Dunn’s test for independent group comparisons, and one-way ANOVA with Sidak’s test for normally distributed data with equal variances.

The statistical tests performed are detailed in the figure legends. Graphs show mean values obtained with n technical or biological replicates, and error bars in all figures represent standard deviation (SD), unless indicated otherwise. A p-value <0.05 has been used as level of significance.

## 3 Results

### 3.1 Identification of an optimal target binder

**Figure 1:**
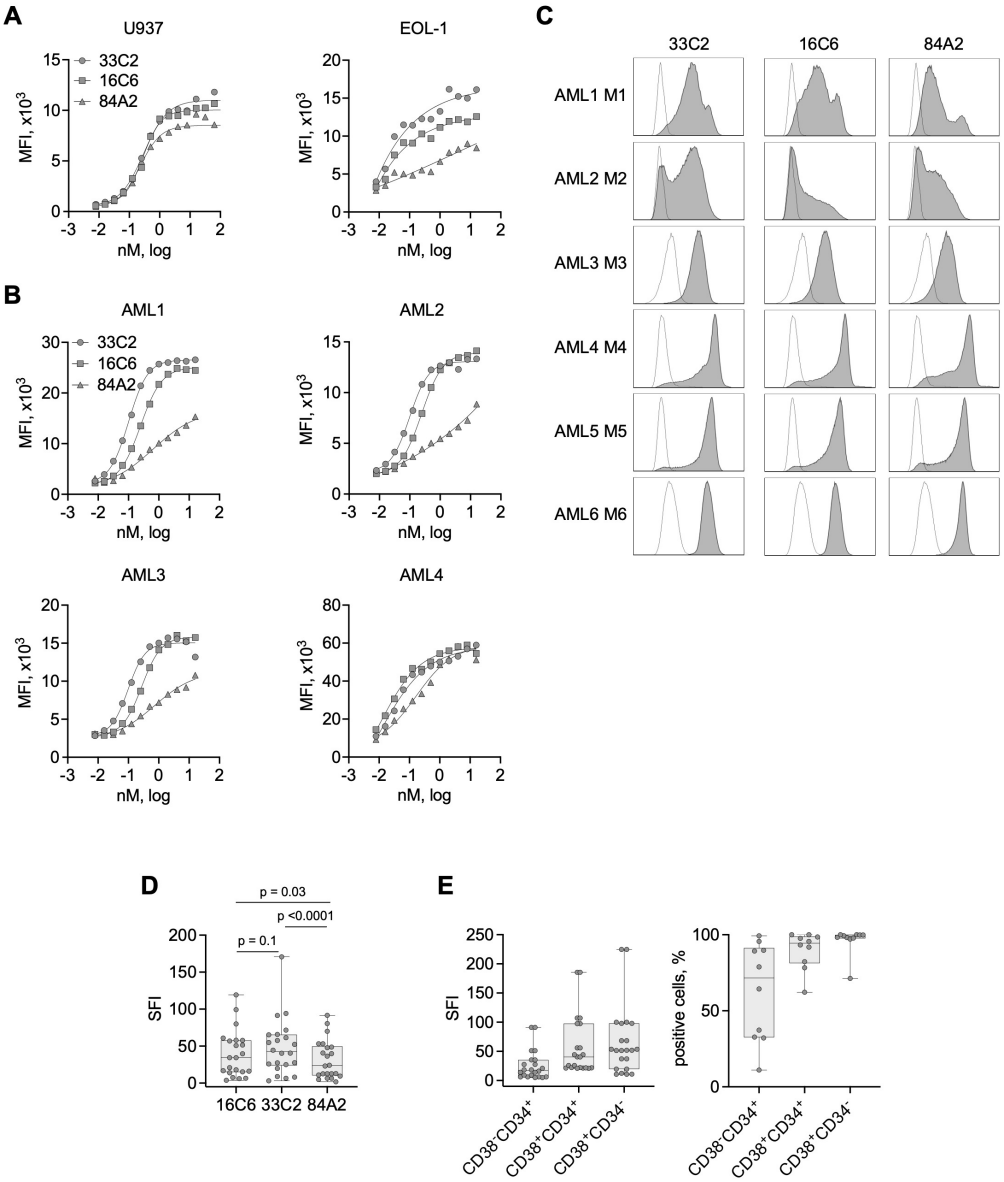
Binding characteristics of CLEC12A antibodies with cell lines and primary AML cells. **(A)** The indicated AML cell lines were incubated with increasing concentrations of three murine CLEC12A mAbs (33C2, 16C6 and 84A2) followed by anti-mouse PE conjugate and flow cytometric analysis. Surface expression is shown as median fluorescence values (MFI). **(B)** Four primary AML patient samples (UPN 11, 20, 10, 15) were stained and analyzed as described in **(A)**. **(C)** Exemplary histograms obtained upon analyzing primary AML cells (UPN 4, 20, 21, 6, 5, 8) of different FAB-subtypes with the mAb clones 33C2, 16C6 and 84A2 (1 µg/ml each) followed by anti-mouse PE and flow cytometric analysis (grey peaks, CLEC12A; outline peaks, isotype control). **(D)** CLEC12A expression on blasts of AML patients (n = 22) as determined with the indicated mAbs. SFI values were calculated by dividing median fluorescence values obtained with specific mAb by median fluorescence values obtained with isotype control; dots: results of individual patients, box: first to third quartiles, whiskers: min-max. P values were determined with Friedman test with Dunn’s multiple comparison test. **(E)** CLEC12A expression on leukemic subpopulations: LSC (CD38^-^CD34^+^), progenitors (CD38-CD34^+^) and mature blasts (CD38^-^CD34^+^) (n=10) detected using the 33C2 mAb. Data are summarized as SFI values (left) and percent of positive cells.

As a first step, we compared the binding properties of 3 murine CLEC12A mAbs termed 33C2, 16C6 and 84A2. Flow cytometric analysis using the CLEC12A-expressing cell lines U937 and EOL-1 revealed that all three mAbs reached saturated binding at approximately 1 nM (0,15 µg/ml) (**Figure 1A**). Saturation was also observed at comparable concentrations on the surface of patient leukemic blasts (**Figure 1B**). Antibody 84A2 always showed the weakest binding, clones 33C2 and 16C6 displayed similar binding characteristics using the cell line U937 and primary AML samples (**Figures 1A, B**). 33C2 achieved slightly higher binding with EOL-1 cells (**Figure 1A**) and had a lower half-maximal binding concentration in titration experiments with the primary samples AML1, 2 and 3 (**Figure 1B**). To ascertain selection of a target binder with optimal characteristics, we expanded our analysis to a panel of 22 AML patient samples representing different FAB-subtypes. The clinical characteristics of the patients are given in **Table 1**. Leukemic cells in all samples were found to be CLEC12A-positive as defined by a specific fluorescence intensity (SFI) level (mean fluorescence intensities measured with specific antibody divided by mean fluorescence intensities obtained with isotype control) of >1.5 (**Figures 1C, D**). Our results confirm earlier data on widespread expression of the antigen in AML. In agreement with our titration experiments, use of mAb 84A2 reproducibly resulted in lower SFI values than the two others mAbs, especially with leukemic cells expressing low CLEC12A levels (**Figure 1C**, AML1 and AML2). The analysis with more than 20 patient samples further revealed that 16C6 showed intermediate binding intensity, whereas 33C2 displayed the highest SFI values (mean SFI=35, 42.9 and 53.9 respectively) (**Figure 1D**). Based on these results, 33C2 was selected as lead candidate.

**Table 1:** Clinical characteristics of the patient cohort.

	FAB	Age (y)	Sex	WHO	NCCN	WBC [giga/l]	Hb [g/dl]	Plt [giga/l]
UPN1	M2	71	F	2	2	282,3	5,7	68
UPN2	M5	71	M	1	0	73,5	13,2	25
UPN3	M5	23	M	4	1	153,5	6,7	44
UPN4	M1	76	F	1	0	10,7	7,4	145
UPN5	M5	68	M	1	1	148,7	9,1	134
UPN6	M4	54	F	1	1	17,2	10,6	167
UPN7	M0	49	M	4	1	29,3	12,9	39
UPN8	M6	70	M	4	1	190,9	7,1	65
UPN9	M1	40	M	1	2	81,3	10,8	51
UPN10	M5	73	M	4	1	90,3	8,9	79
UPN11	M1	83	W	0	0	121	7,5	15
UPN12	M2	64	M	0	0	0	0	0
UPN13	M5	25	M	NA	NA	NA	NA	NA
UPN14	M4	76	F	NA	NA	NA	NA	NA
UPN15	M5	70	M	0	0	42,5	9,7	80
UPN16	M5	90	M	4	3	87,3	9	105
UPN17	M4	41	F	1	2	112,7	8,5	30
UPN18	M4	21	M	1	0	125	10,9	24
UPN19	M2	63	M	1	0	183,3	8,1	31
UPN20	M2	76	W	1	1	140,9	12	70
UPN21	M3	29	M	1	0	21,6	7,1	61
UPN22	M2	88	M	4	NA	49	7,9	95

UPN, unique patient number; FAB, French–American–British classification; WHO, WHO Classification 2008; 1—AML with recurrent genetic aberrations; 2—AML with myelodysplasia-related changes; 3—Therapy-related myeloid neoplasms; 4 AML, not otherwise specified; NCCN, National Comprehensive Cancer Network classification 2018; 0—favorable risk, 1—intermediate risk, 2—poor risk, 3—not defined; WBC, white blood cell count; Plt, thrombocytes; NA, no data available.

Next, we tested binding of 33C2 to different leukemia cell subpopulations, i.e. leukemic stem cells (LSC, CD34^+^CD38^-^), progenitor cells (CD34^+^ CD38^+^) and CD34^-^, CD38^+^ leukemic blasts. We detected substantial CLEC12A positivity over all subpopulations in all 22 patient samples (SFI>5), albeit the LSC population displayed lower SFI values and less positive cells when compared with the two other populations (**Figure 1E**).

### 3.2 Generation of IL-15 immunocytokines and induction of NK cell reactivity against CLEC12A-expressing cell lines

**Figure 2:**
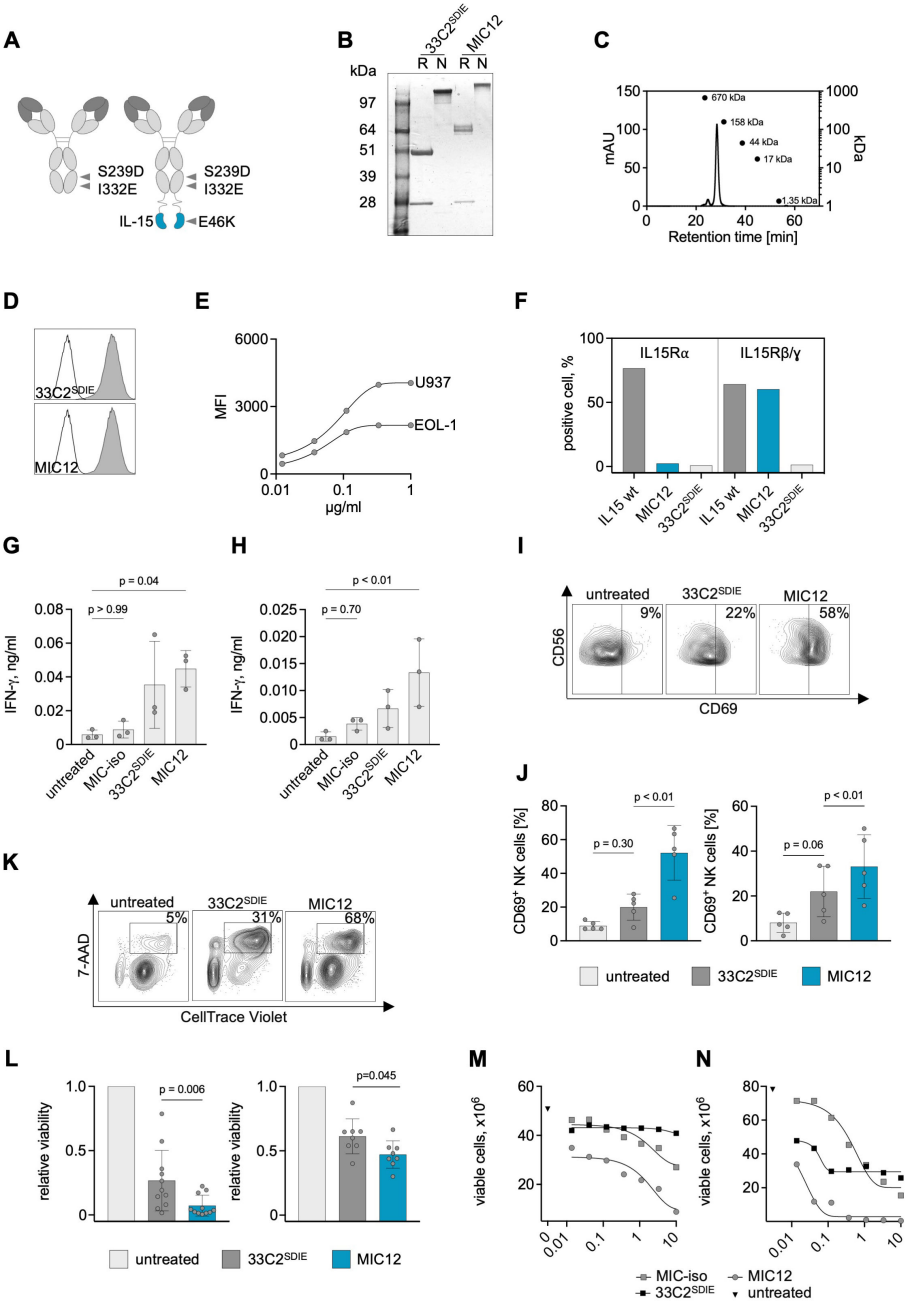
Production of anti-CLEC12A IL-15 immunocytokines and characterization of on-target and off-target activity using leukemia cell lines. **(A)** Schematic illustrations of the MIC12 immunocytokine consisting of the humanized VH and VL domains of the 33C2, human IgG1 constant domains containing the SDIE modification (S239D; I332E), (G_4_S)_4_ linker and a C-terminal IL15^E46K^ mutein (right) and a control Fc-optimized mAb without IL-15 moiety (33C2^SDIE^) (left). **(B)** SDS-PAGE electrophoresis of the humanized Fc-optimized 33C2^SDIE^ antibody and MIC12 in reducing (R) and non-reducing (N) conditions. Expected molecular weights: 23 kDa for the light chain, 63 kDa for the heavy chain of the MIC construct and 49 kDa for the heavy chain of the antibody. **(C)** Exemplary results of a size exclusion chromatography of the MIC12. Expected size of the full construct 172 kDa. **(D)** Binding of MIC12 and 33C2^SDIE^ to U937 cells at equimolar concentrations detected with donkey anti-human IgG-PE conjugate and flow cytometric analysis (grey peaks, anti-CLEC12A; outline peak, donkey anti-human IgG-PE conjugate). **(E)** Dose titration of MIC12 on U937 and EOL-1 cells detected with donkey anti-human IgG-PE conjugate and flow cytometric analysis. **(F)** Binding of MIC12 to IL-15Rα-expressing TF-1 (left) and IL15Rβ/γ-expressing M-07e cells (right). Binding was detected as described in **(D)**. A MIC construct with irrelevant specificity containing wild-type IL-15 was used as a positive control, 33C2^SDIE^ was used as a negative control. **(G, H)** PBMC from healthy donors (n=3) were cocultured with U937 **(G)** or EOL-1 **(H)** cells (E:T 2.5:1) with the indicated constructs at equimolar concentrations (1 µg/ml for the antibody and 1.2 µg/ml for the MIC). IFN-γ secretion in culture supernatants was determined after 24 h using ELISA. MIC-iso, – MIC construct with irrelevant specificity. P values were determined with Kruskal-Wallis test, followed by Dunn’s multiple comparison test. **(I, J)** PBMC from healthy donors were incubated with U937 and EOL-1 cells at an E:T ratio of 2.5:1 in the presence of constructs for 72h (1 µg/ml for the antibody and 1.2 µg/ml for the MIC). CD69 expression on CD3-CD56+ NK cells was determined by flow cytometry. **(I)** Exemplary flow cytometry plots derived with U937 target cells; the percentage of CD69^+^ NK cells is indicated. **(J)** Combined results obtained with 5 PBMC donors using U937 (left) and EOL-1 cells (right). Data are shown as means of triplicate measurements. P values were determined with Kruskal-Wallis test, followed by Dunn’s multiple comparison test. **(K, L)** PBMC from healthy donors were incubated for 72 h with CellTrace Violet (CTV)-labelled U937 or EOL-1 cells at an E:T ratio of 2.5:1 in the presence of the indicated constructs at equimolar concentration (1 µg/ml for the antibody and 1.2 µg/ml for the MIC). Target cell lysis was determined by flow cytometry. **(K)** Exemplary flow cytometry plots obtained with U937 target cells; the percentage of dead target cells (CTV^+^7AAD^+^) is indicated. **(L)** Combined results obtained with U937 (left) and EOL-1 (right) cells using 11 and 7 independent PBMC donors, respectively. Cell viability was normalized to the untreated control (mean of duplicate wells). P values were determined with Friedmans test, followed by Dunn’s multiple comparison test. **(M, N)** PBMC **(M)** or purified NK cells **(N)** from healthy donors were incubated for 72 h with CellTrace Violet (CTV)-labelled U937 cells at an E:T ratio of 2.5:1 (PBMC) or 1:1 (NK cells) in the presence of the indicated constructs titrated in a broad concentration range. Target cell lysis was determined by flow cytometry. Representative results from a total of three donors with similar outcomes are presented.

To generate a MIC protein targeting CLEC12A (MIC12), the construct was composed of humanized V_H_ and V_L_ domains of the 33C2 antibody, the Fc domain of human IgG1 with SDIE modification and a human IL-15^E46K^ mutein with abrogated binding to IL-15Rα as described in the Methods section (**Figure 2A**). In addition, an Fc-optimized antibody without IL-15 moiety (33C2^SDIE^) was cloned as a control. In order to gain a first understanding regarding the producibility of the MIC constructs, we evaluated purity and aggregate content using SDS-PAGE and size exclusion chromatography, which documented that our MIC construct displayed the expected molecular weight and showed high purity and no aggregates (**Figures 2B, C**). Staining of U937 and EOL-1 cells with MIC12 confirmed that target binding was not affected by humanization and immunocytokine construction (**Figures 2D, E**). Next, we validated binding of the MIC construct to IL-15 receptors. As expected, our MIC12 construct containing the E46K-mutein that abrogates IL15Rα-binding exhibited very weak interaction with IL-15Rα-expressing TF-1 cells compared to an IL-15^WT^ control construct, but retained binding to IL15Rβ/γ-expressing M07e cells (**Figure 2F**).

Given that exogenous cytokines and so far available immunocytokines suffer from drawbacks like Treg induction in case of IL-2 and systemic toxicity upon higher dosing in case of both IL-2 and Il-15 (15–17), we have developed an IL-15 moiety with targeted activity to reduce nonspecific effector cell activation and cytokine release, a key feature of our recently published MIC platform (25). To evaluate this property for MIC12, we analyzed how our construct influenced IL-15-mediated NK cell activation. As control, we used a construct in which the 33C2 variable domain was replaced by an irrelevant target binder (MOPC-21, MIC-iso). We performed a comparative analysis of MIC-iso with MIC12 regarding their ability to stimulate IFN-γ secretion in cocultures of PBMC from healthy donors (PBMC) using U937 and EOL-1 cells as targets. This experiment documented that MIC-iso induced no relevant IFN-γ secretion, whereas target recognition by 33C2^SDIE^ and MIC12 induced significant NK cell reactivity (**Figures 2G, H**), confirming previous findings on the target cell-restricted mode of action of MIC constructs (25).

To assess the benefits of the MIC12 construct over the Fc-optimized CLEC12 mAb, we first cocultured PBMC with U937 and EOL-1 cells and quantified NK cell activation and cytotoxicity. MIC12 was clearly superior to 33C2^SDIE^ in inducing CD69 expression on CD3^-^CD56^+^ NK cells with both target cell lines (**Figures 2I, J**). This was mirrored by significantly more pronounced target cell lysis as detected in flow cytometry-based cytotoxicity assays with multiple PBMC donors (**Figure 2K, L**). Of note, effects of both, 33C2^SDIE^ and MIC12, against EOL-1 were generally weaker compared to U937. Analysis of target antigen expression revealed that U937 express profoundly more CLEC12A, which correlates with substantially higher efficacy upon therapeutic targeting (**Figure 2E**).

Dose titration experiments using both PBMC and isolated NK cells confirmed the superior therapeutic efficacy of MIC12 compared to the Fc-optimized CLEC12A mAb over a broad range of concentrations and also documented a wide therapeutic window upon comparison of MIC12 and MIC-iso (**Figures 2M, N**).

Next, we aimed to exclude that MIC12 altered NK cell viability, e.g. by inducing fratricide. To this end, we cultured purified NK cells without target cells in the presence of the MIC12 or the 33C2^SDIE^ antibody and employed flow cytometric analysis with 7-AAD to confirm that treatment did not affect NK cell viability (**Supplementary Figure S1**).

### 3.3 MIC12 induces NK cell proliferation and long-term clearance of target cells

**Figure 3:**
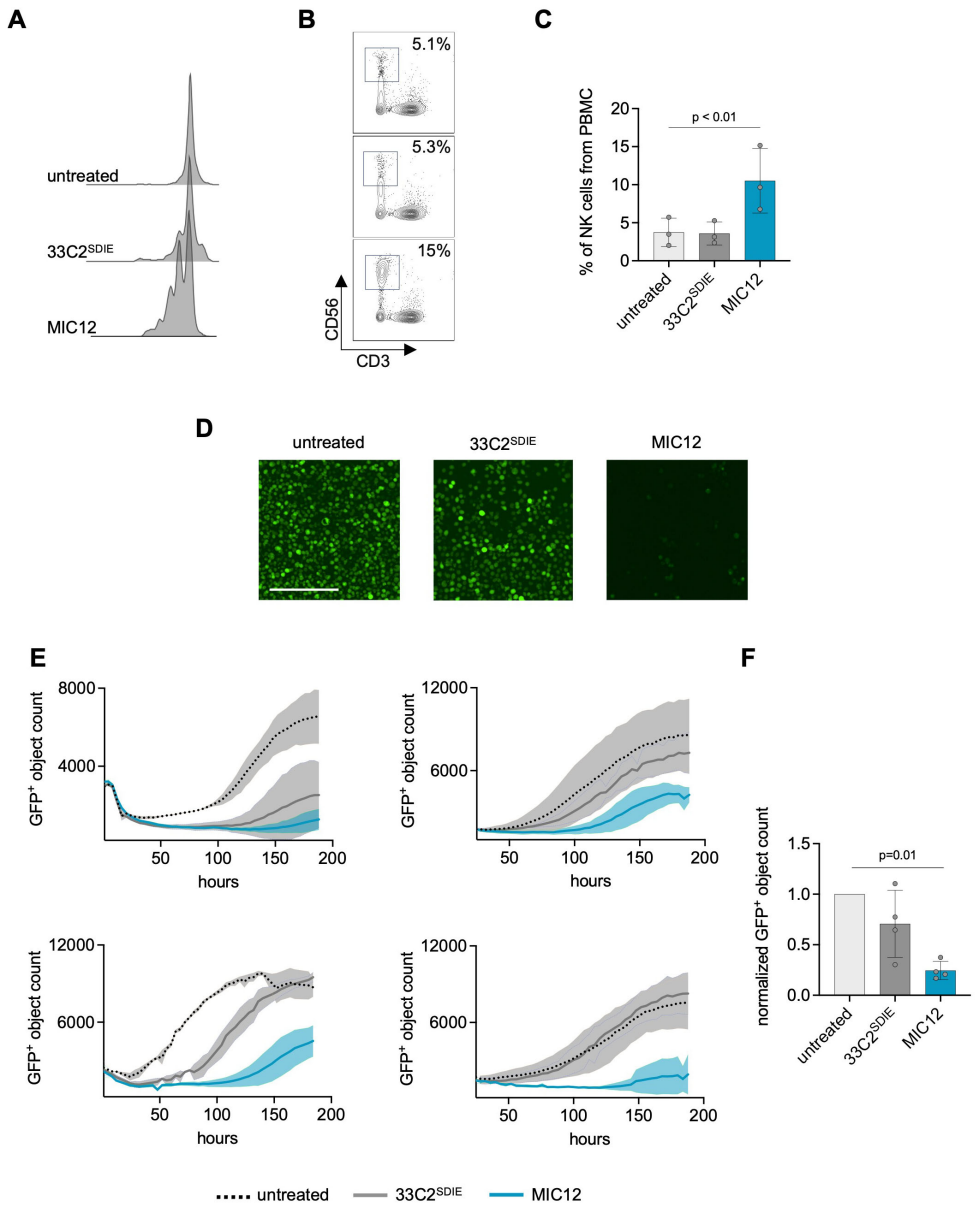
MIC12 induces NK cell proliferation and mediates superior long-term efficacy against U937 cells. **(A-C)** CTV-labelled PBMC were cultured with U937 cells (E:T 2.5:1) and the indicated constructs at equimolar concentrations. Fresh target cells and constructs were added to the wells after 3 days followed by continued incubation for additional 4 days. **(A)** CTV dye dilution was analyzed by flow cytometry. Exemplary histogram plots of one representative experiment are shown. **(B)** Exemplary dot plots demonstrating expansion of CD56^+^ NK cells. Top, untreated; middle, 33C2^SDIE^; bottom, MIC12. **(C)** Quantification of NK cell expansion after 8 days of incubation with the indicated constructs at equimolar concentrations, presented as percentage of the total PBMC population. Each data point represents data obtained with a single PBMC donor. **(D, E)** Long-term cytotoxicity assay. PBMC were incubated 8 days with GFP^+^ U937 cells (E:T 2.5:1) and indicated treatments (1 µg/ml). Target cell viability was quantified using Incucyte S3. **(D)** Exemplary microscopic images, scale bar: 200 µm. **(E)** Growth curves representing data from cocultures with four independent PBMC donors. Shown are GFP^+^ object counts at each timepoint with mean values derived from two wells. Standard deviations are shown as shaded areas. **(F)** Combined results obtained with 4 PBMC donors, 120 hours after assay initiation. Viable cell counts were normalized to untreated control (mean of triplicate measurements). P-values were determined using the Kruskal-Wallis test followed by Dunn’s multiple comparison test.

Since ADCC-inducing antibodies suffer from a low capacity to induce NK cell proliferation, we aimed to overcome this limitation with our MIC construct. To this end we compared NK cell proliferation upon treatment with Fc-optimized mAb and MIC12. Flow cytometric dye dilution assays showed progressively dimmer CTV peaks in the CD3^-^CD56^+^ NK cell population upon treatment with MIC12 compared to Fc-optimized mAb after 7 days of coculture with target cells, which is clearly indicative of NK cell proliferation (**Figure 3A**). Quantification of NK cells in cultures revealed that, unlike the corresponding Fc-optimized mAb, MIC12 promoted pronounced NK cell proliferation, as revealed by more than doubling the NK cell population within PBMC (**Figures 3B, C**).

Next, we determined whether the MIC12-induced expansion of effector cells over time translated into improved long-term efficacy. To this end, we cocultured GFP-expressing U937 cells with PBMC in the presence of the constructs and monitored growth of target cells over one week by live cell imaging. As evident from the exemplary images (**Figure 3D**), the Fc-optimized antibody achieved only marginal effects, whereas treatment with MIC12 resulted in almost complete depletion of target cells. This was mirrored in time course quantification of target cell density using PBMC of four independent donors (**Figure 3E**) and in the cumulated data of all 4 donors after 120h (**Figure 3F**). Despite a substantial donor variation, in all cases target cells treated with 33C2^SDIE^ demonstrated either outgrowth after initial short-term depletion (donors 1-3) or were not affected at all (donor 4). In stark contrast, MIC12 mediated prolonged suppression of target cell growth. Notably, all effects observed following MIC12 treatment were NK cell-dependent, as no T cell activation (**Supplementary Figure S2A**), cytokine secretion (**Supplementary Figure S2C**), T cell-mediated cytotoxicity (**Supplementary Figures S2D, E**), or proliferation (**Supplementary Figure S2G**) were detected.

These findings showcase the remarkable superiority of our MIC construct over Fc-optimized mAb in eliciting NK cell activation, proliferation, and sustained antitumor efficacy.

### 3.4 MIC12 induces potent NK cell reactivity against primary AML cells

**Figure 4:**
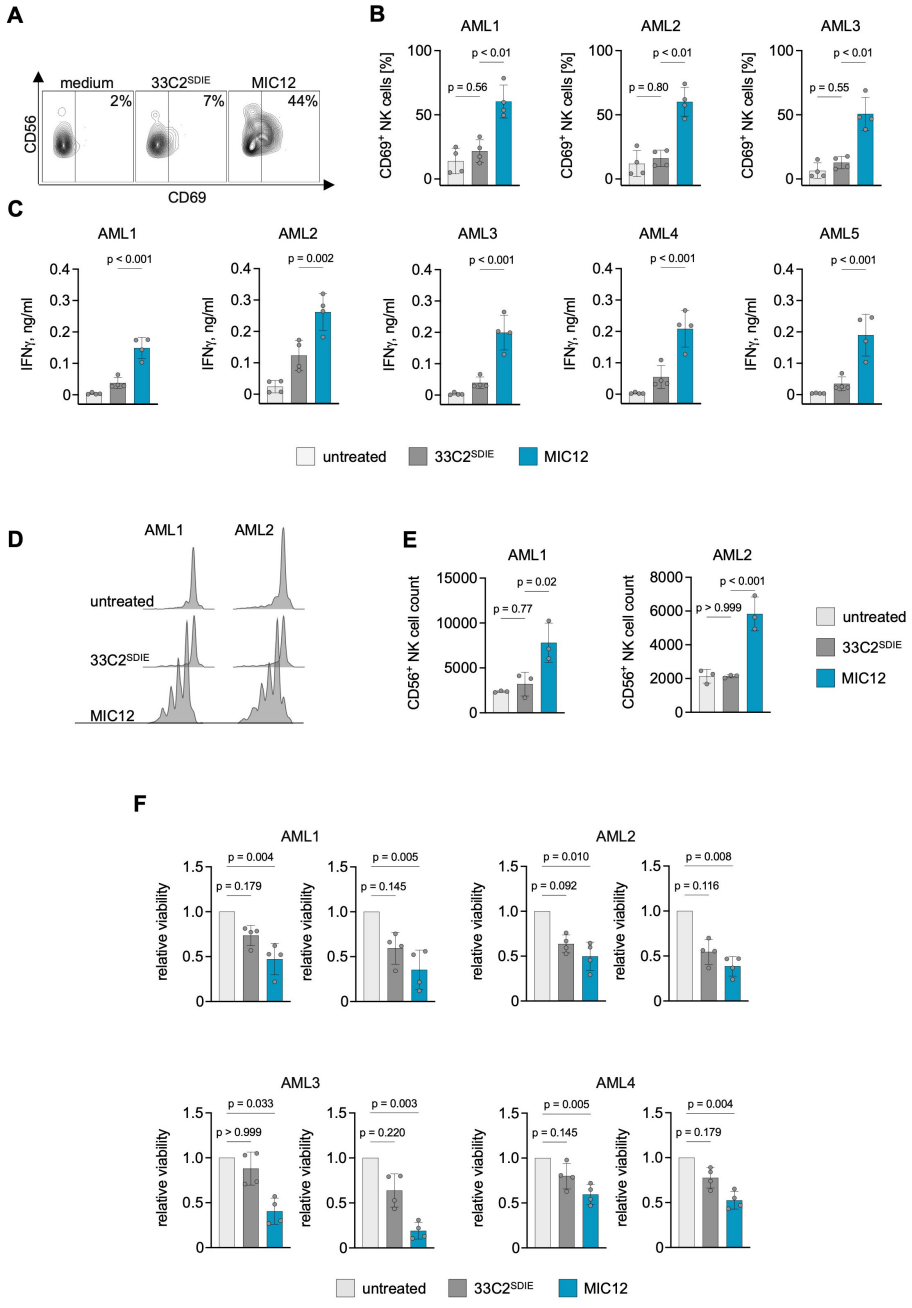
MIC12 induces superior NK cell reactivity against patient AML cells. **(A, B)** PBMC of healthy donors were incubated with patient AML cells (E:T 2.5:1) in the presence or absence of the indicated at equimolar concentrations. After 3 days, CD69 expression on CD3^-^CD56^+^ NK cells was assessed using flow cytometry. **(A)** Exemplary flow cytometry plots, the percentage of CD69^+^ NK cells is indicated. **(B)** Combined results obtained with 4 independent PBMC donors incubated with 3 different AML patient samples. P values were determined with One way ANOVA, followed by Sidak’s multiple comparison test. **(C)** PBMC from 4 donors were each incubated with leukemic cells of 5 different AML patients. After 24 hours, IFN-γ levels in culture supernatants were measured by ELISA P values were determined with One way ANOVA, followed by Sidak’s multiple comparison test. **(D, E)** Proliferation of CTV-labelled NK cells incubated for 6 d with patient AML cells in the presence of the indicated constructs at equimolar concentrations (0,1 µg/ml for the antibody and 0,12 µg/ml for MIC) was assessed by flow cytometry. **(D)** Exemplary dye dilution curves are shown with 2 AML samples. **(E)** Quantification of CD3^-^CD56^+^ NK cells in cocultures combining PBMC from three independent donors with samples from two different AML patients. P values were determined with one-way ANOVA with Sidak’s multiple comparison test. **(F)** PBMC of 4 different donors were cultured with CTV-labelled patient AML cells at E:T ratios of 20:1 (left) and 10:1 (right) for 6 days. Viability of AML cells was determined by flow cytometry. Shown are viable cell counts normalized to untreated control. P values were determined with Kruskal-Wallis test, followed by Dunn’s multiple comparison test.

Finally, we aimed to determine whether superior efficacy of MIC12 could also be demonstrated with primary AML cells from leukemia patients. To this end, we analyzed NK cell activation and IFN-γ release in cocultures of PBMC with primary AML blasts. Flow cytometry analysis revealed profound CD69 upregulation as marker for activation upon treatment with MIC12, but not with 33C2^SDIE^, and this held true for combinations of leukemic cells of three AML patients and four PBMC donors (**Figures 4A, B**). Furthermore, MIC12 promoted IFN-γ secretion to levels markedly exceeding those induced by 33C2^SDIE^ (**Figure 4C**).

To ensure that the key advantage of our MIC constructs – induction of NK cell proliferation – was also achieved when using primary patient samples, we employed dye dilution assays in six-day cocultures of CTV-labeled PBMC and patient AML cells. The observed multiple CTV intensity peaks revealed robust NK cell proliferation induced by MIC12, contrasting with the unchanged CTV staining intensity in cultures treated with 33C2^SDIE^ (**Figure 4D**). Quantitative analysis revealed a pronounced increase in NK cell proliferation following MIC12 treatment, whereas cultures treated with an Fc-optimized antibody remained unaltered (**Figure 4E**, **Supplementary Figure S3**). Within the PBMC, a 3- to 10-fold NK cell expansion clearly exceeding the range of physiological NK cell percentages in healthy individuals was detected, which emphasizes the biological relevance of the induced proliferation (**Supplementary Figure S3**). Similarly to the experiments with cell lines, MIC12 did not induce T cell activation (**Supplementary Figure S2B**) or proliferation (**Supplementary Figure S2G**) in cocultures with primary AML cells. Finally, we studied the anti-leukemic activity of the MIC12 and 33C2^SDIE^ in long-term cytotoxicity assays with the patient cells. We cultured 4 primary AML samples with 4 PBMC donors and quantified surviving AML cells by flow cytometry (**Figure 4F**). MIC12 consistently induced profoundly stronger NK cell cytotoxicity against CLEC12A-positive AML blasts as compared to the Fc-optimized antibody which did not achieve statistically significant effects in any combination. This supports the essential role of IL-15 in boosting anti-leukemic reactivity.

## 4 Discussion

Here we report on the development and preclinical characterization of immunocytokines with conditional IL-15 activity targeting CLEC12A for AML treatment (MIC12). This molecule expands our previously described immunocytokine platform, characterized by NK cell-focused IL-15 activity, which has shown superior efficacy compared to Fc-optimized antibodies while reducing systemic cytokine effects to AML.

CLEC12A (CLL-1) emerges as a promising antigen for AML immunotherapy. Preclinical studies have demonstrated the efficacy of CLEC12A bispecific antibodies (30, 31), antibody-drug conjugates (32, 33), antibody-cytokine fusion proteins (34) and CLEC12A-targeted CAR-T cells (35) in eliminating AML cells *in vitro* and *in vivo*. Several clinical trials evaluating safety and efficacy of CLEC12A-targeting bispecific antibodies (NCT03038230), CAR-T cells (NCT04219163, NCT06128044, NCT06017258) and antibody-drug conjugates a have been recently completed or are currently ongoing and show manageable toxicity and signs of efficacy (36, 37). A major obstacle of antibody-based immunotherapy is the unintended targeting of healthy tissues expressing the antigen, leading to side effects. CLEC12A is an attractive target for immunotherapeutic approaches due to its limited expression on normal hematopoietic stem cells and other healthy tissues but widespread prevalence on AML blasts and leukemic stem cells (38), which is confirmed by our data obtained in a cohort of 22 AML patients (**Figures 1D, E**). Our MIC molecules with IL-15 activity being restricted to target antigen binding, combined with CLEC12A’s rather tumor-restricted expression, provides a promising and potentially safe approach for AML therapy.

In recent years many immunocytokines demonstrated remarkable preclinical efficacy, including one CLEC12A-targeting IL15-based NK cell engager (34). However, in all these immunocytokines, the activity of the cytokine moieties is independent of antibody target binding. As a result, these molecules fail to exclusively focus the cytokine effects to the tumor site, leading to profound systemic off-target effects and dose-limiting toxicity (19–21). This is due to the fact that only a minor fraction of the applied immunocytokine is bound to the target at any given time, while the majority is distributed systemically where it mediates undesired effects (39). Furthermore, the cytokine moiety can drive localization of the drug to the receptor-expressing cells irrespectively of antibody specificity (40). So far only few attempts were undertaken to limit the stimulatory activity of the cytokine to the tumor site. For example, an attenuated IL-21 mutein was fused to an PD-1 antibody to restrict cytokine activity to PD-1-expressing cells (41). An IL-2 variant temporally inactivated by a linker that is cleavable exclusively by tumor-specific proteases was used to restrict cytokine function to tumor microenvironment enriched with specific proteolytic enzymes (42). Finally, *de novo* designed IL2/15Rβγ agonists without binding to IL2Rα showed improved activation of CD8^+^ T cells, reduced expansion of CD4^+^ Tregs and improved targeting to the tumor site (43). The design of our MIC immunocytokines incorporates an IL-15^E46K^ mutein with disrupted IL-15Rα binding, allowing for substitution of trans-presentation by target antigen binding, which facilitates target-restricted IL-15Rβγ activation (25). Similar to other MIC of our platform, MIC12 exhibited pronounced target specificity, as indicated by CLEC12A-dependent IFN-γ release (**Figure 2H**). Although further studies in animal models and ultimately patients are needed to fully validate our concept with regard to potential toxicity/safety, our data provide support for IL-15^E46K^-based Fc-optimized MIC as a promising immunocytokine format with conditional IL-15 activity.

The pivotal role of NK cells in mediating ADCC upon application of antitumor mAbs is well established. Current efforts are focused on enhancing efficacy by optimizing interactions with activating Fc receptors. The SDIE modification represents a key advance in boosting NK cell-mediated ADCC, and several Fc-optimized mAbs with this modification are either in clinical evaluation or have recently gained approval, such as the CD19-targeting tafasitamab. Combining Fc optimization with IL-15 activity further amplifies the therapeutic potential. Alike our IL-15^E46K^-based Fc-optimized immunocytokine constructs targeting CD20 and CD19 (25), MIC12 achieved superior NK cell activation and killing of cancer cell lines and primary AML cells when compared with Fc-optimized antibody. Notably, NK cells are generally scarce within PBMC and have been reported to be functionally impaired in MDS and AML patients due to an immunosuppressive microenvironment and chemotherapy (44–46). Therefore and given the unfavorable effector to target ratios that prevail in most patients, NK cell-stimulating therapeutic mAbs so far fail to achieve disease control. Thus, expansion of NK cells and maintenance of their functional capacity is key for successful therapy. Administration of IL-15 leads to massive NK cell expansion in animal models and cancer patients (47–49). In comparative analyses with MIC12, an Fc-optimized control mAb was unable to trigger NK cell expansion, emphasizing the combinatorial effect of Fc receptor engagement and IL-15 signaling in a targeted manner achieved by our constructs. The observed synergy is in agreement with the enhancement of IL-15-mediated NK cell expansion *in vivo* by FcR signaling (50). This effect can be explained by the recently identified crosstalk between IL-15-induced STAT-5 signaling and ITAM-dependent activating receptors (such as FcγRIII) (51, 52). The NK cell proliferation induced by MIC12 translated into sustained long-term control of U937 cell growth, contrasting with the limited impact of 33C2^SDIE^. This was mirrored in long-term killing assays with primary AML cells, where 33C2^SDIE^ failed to produce statistically significant effects, but MIC12 effectively depleted AML blasts, further emphasizing the potential of MIC12 to improve leukemia cell elimination in AML patients.

Of note, NK cells demonstrated promising therapeutic potential in AML upon adoptive transfer (44, 53, 54), but the transferred NK cells reportedly become functionally impaired (55). It is tempting to speculate that, beyond being applied as single agent, MIC12 may ensure maintenance of transplanted NK cell upon combinatorial application.

In summary, our findings establish MIC12^SDIE^ as a reagent with superior antileukemic activity, enabled by NK cell proliferation induced by target cell-restricted IL-15 activity that surpasses the potency of Fc-optimized monoclonal antibodies and thus constitutes a promising novel compound for AML therapy.

## Data Availability

The original contributions presented in the study are included in the article/**Supplementary Material**. Further inquiries can be directed to the corresponding author.
