# Supplementary material for: CLEC12A-directed immunocytokine with target cell–restricted IL-15 activity for treatment of acute myeloid leukemia
Source: Front Immunol. 2025 Mar 27;16:1561823. doi: 10.3389/fimmu.2025.1561823 (PMC11983603; doi:10.3389/fimmu.2025.1561823)
Supplement: Supplementary file 1 [file DataSheet1.pdf]

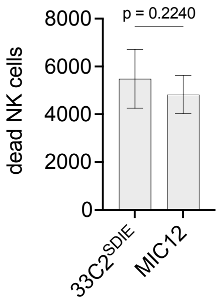


# *Supplementary Figures*

**Supplementary figure S1. MIC12 does not induce NK cell fratricide.**

Purified NK cells from two healthy donors were incubated in the presence of MIC12 (1.2 µg/ml) for 72h. 33C2$^{SDIE}$ served as a negative control. NK cell viability was quantified by flow cytometry using 7AAD staining and CD3 CD56 counterstaining.

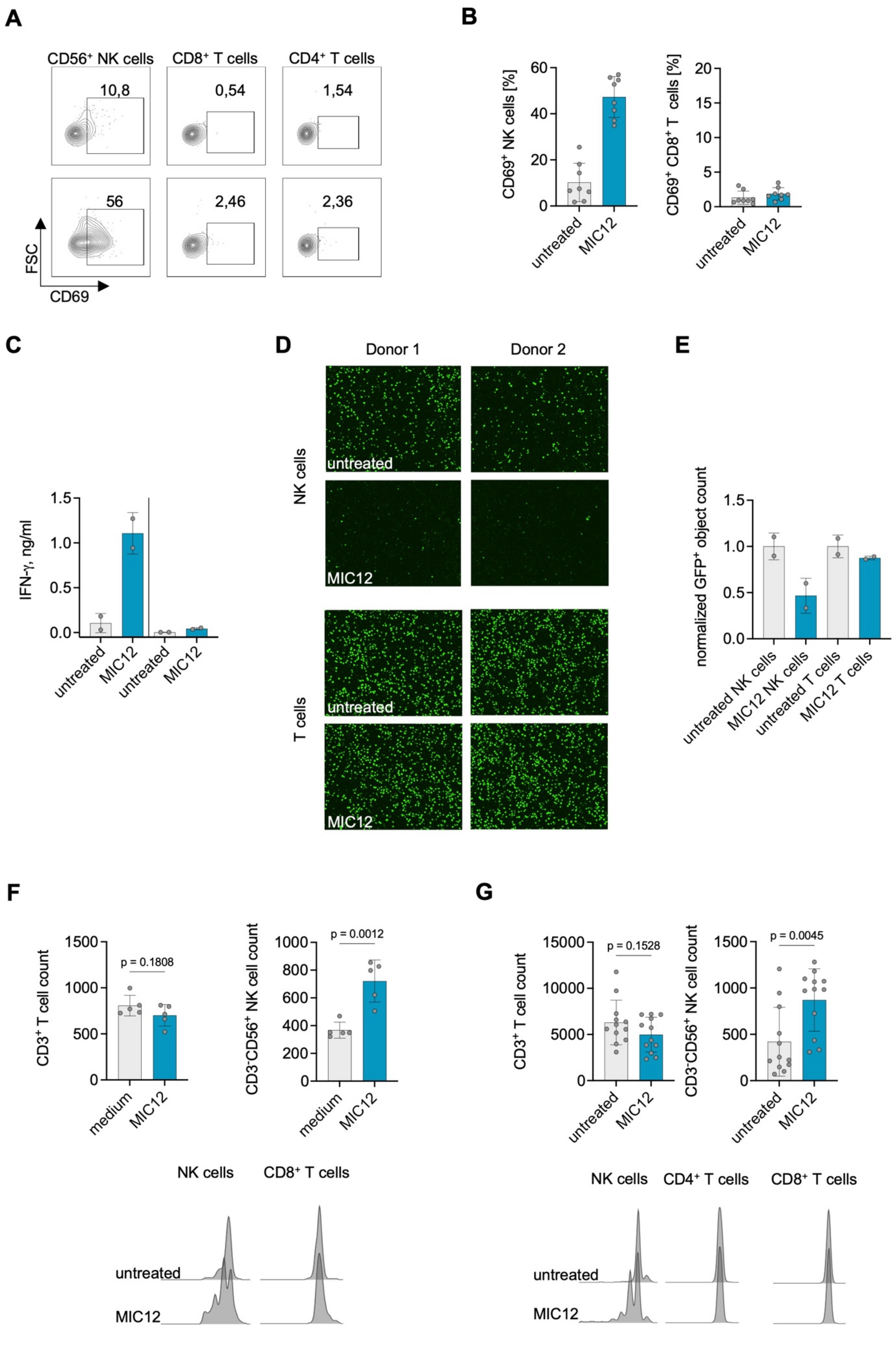
A
CD56⁺ NK cells
CD8⁺ T cells
CD4⁺ T cells
10,8
0,54
1,54
56
2,46
2,36
FSC
CD69
B
CD69⁺ NK cells [%]
CD69⁺ CD8⁺ T cells [%]
untreated
MIC12
C
IFN-γ, ng/ml
untreated
MIC12
untreated
MIC12
D
Donor 1
Donor 2
NK cells
T cells
untreated
MIC12
untreated
MIC12
E
normalized GFP⁺ object count
untreated NK cells
MIC12 NK cells
untreated T cells
MIC12 T cells
F
CD3⁺ T cell count
p = 0.1808
medium
MIC12
CD3⁻CD56⁺ NK cell count
p = 0.0012
medium
MIC12
NK cells
CD8⁺ T cells
untreated
MIC12
G
CD3⁺ T cell count
p = 0.1528
untreated
MIC12
CD3⁻CD56⁺ NK cell count
p = 0.0045
untreated
MIC12
NK cells
CD4⁺ T cells
CD8⁺ T cells
untreated
MIC12

**Supplementary figure S2. Impact of MIC12 on T cell activation, proliferation and cytotoxicity.**

(A) PBMC from healthy donors were incubated with U937 cells at an E:T ratio of 2.5:1 in the presence of MIC12 (1.2 μg/ml) constructs for 72h. Exemplary flow cytometry plots showing CD69 expression on $CD3^{-}CD56^{+}$ NK cells $CD3^{+}CD4^{+}$ and $CD3^{+}CD4^{+}$NK cells.
(B) Primary AML cells (n=2) were incubated with PBMC from healthy donors (n=4) at an E:T ratio of 10:1 in the presence of 1 μg/ml MIC12 for 72h. CD69 expression on $CD3^{-}CD56^{+}$ NK cells and $CD3^{+}CD8^{+}$ T cells was quantified by flow cytometry.
(C) Isolated NK and T cells from healthy donors (n=2) were cocultured with U937 cells (E:T 2.5:1) with MIC12 (1.2 μg/ml). IFN-γ secretion in culture supernatants was determined after 24 h using ELISA.
(D,E) Cytotoxicity assay with purified NK and T cells. Isolated effector cells from healthy donors (n=2) were incubated 24 h with $GFP^{+}$ U937 cells and MIC12 (1.2 μg/ml). Target cell viability was quantified using Incucyte S3. (D) Exemplary microscopic images (E) $GFP^{+}$ object counts after 24h normalized to untreated condition, mean values derived from three wells.
(F,G) Proliferation of NK and T cells under MIC12 treatment.
Upper panel: Proliferation of CTV-labelled NK cells and $CD3^{+}$ T cells in PBMC cultures incubated for 6 d with U937 cell line (F) and patient AML cells (G) in the presence of 1.2 μg/ml of MIC12. Corresponding populations were quantified by flow cytometry. (F) Representative data from 1 PBMC donor measured in 5 wells are shown. (G) Data from 4 AML samples combined with 3 PBMC donors are shown. P values were determined with Mann-Whitney test.
Lower panel: Exemplary dye dilution curves obtained with U937 cells (F) for NK and cytotoxic T cells (F) and primary AML cells (G) for NK, $CD4^{+}$ and $CD8^{+}$ T cells (G).

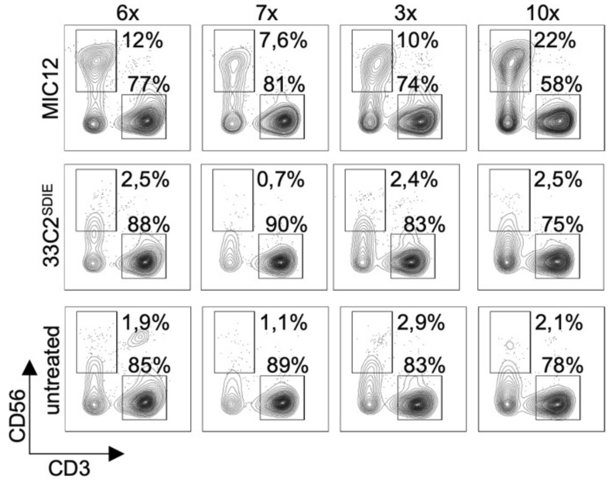


**Supplementary figure S3. NK cell expansion in the presence of MIC12 or 33C2$^{SDIE}$ in cocultures with human AML cells.**

Exemplary flow cytometry plots derived with 4 PBMC donors; the percentage of CD56$^{+}$ NK cells and CD3$^{+}$ T cells and NK cell expansion rates are indicated.

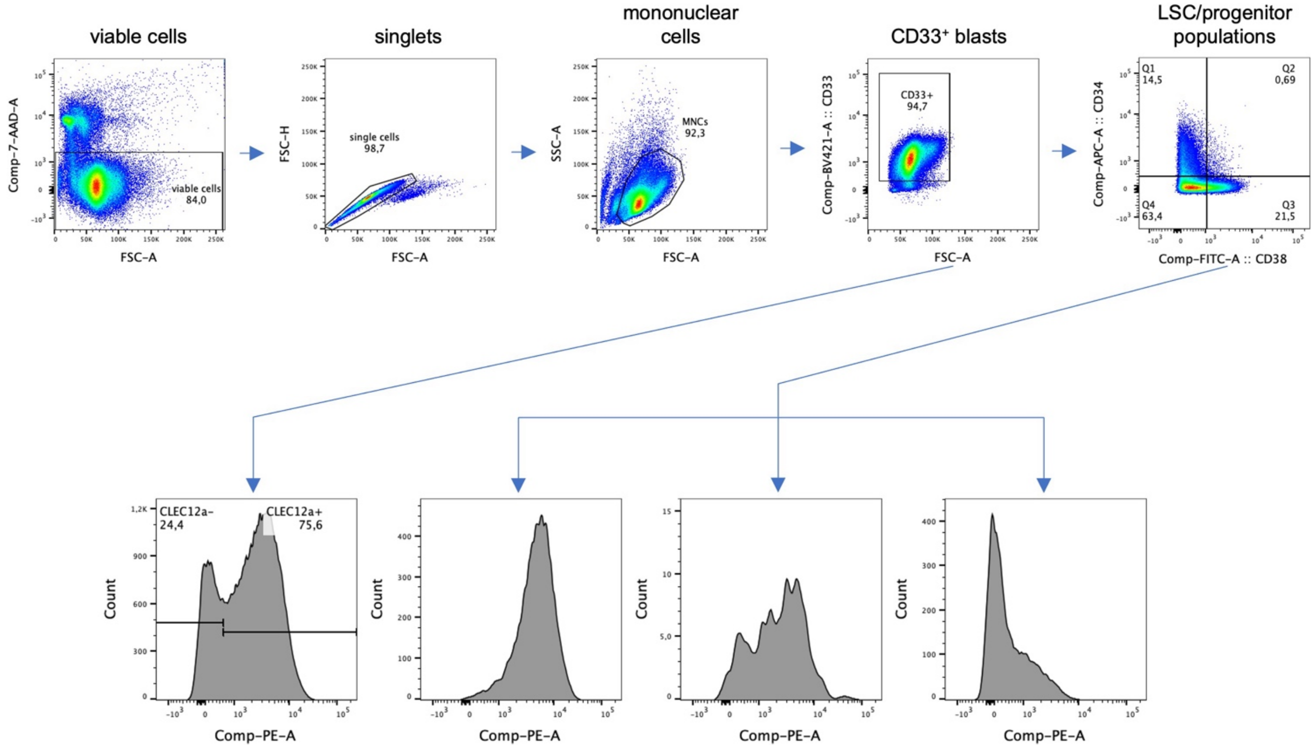


**Supplementary figure S4: Gating strategy for flow cytometry quantification of CLEC12A expression on bulk CD33⁺ AML blasts and LSC/progenitor subpopulations.**

Representative flow cytometry plots illustrate the sequential gating strategy used to analyze CLEC12A expression. AML blasts were identified based on CD33 positivity, followed by gating on LSC/progenitor subpopulations, including $CD38^{-}CD34^{+}$, $CD38^{+}CD34^{+}$, and $CD38^{+}CD34^{-}$ subsets. CLEC12A expression was then assessed within each gated population.
